# Type IV Pili Can Mediate Bacterial Motility within Epithelial Cells

**DOI:** 10.1128/mBio.02880-18

**Published:** 2019-08-20

**Authors:** Vincent Nieto, Abby R. Kroken, Melinda R. Grosser, Benjamin E. Smith, Matteo M. E. Metruccio, Patrick Hagan, Mary E. Hallsten, David J. Evans, Suzanne M. J. Fleiszig

**Affiliations:** a School of Optometry, University of California, Berkeley, California, USA; b Vision Science Program, University of California, Berkeley, California, USA; c Undergraduate Research Apprentice Program, University of California, Berkeley, California, USA; d College of Pharmacy, Touro University California, Vallejo, California, USA; e Graduate Group in Microbiology, University of California, Berkeley, California, USA; f Graduate Group in Infectious Diseases and Immunity, University of California, Berkeley, California, USA; Georgia Institute of Technology School of Biological Sciences

**Keywords:** bacterial exit, bacterial motility, epithelial cells, intracellular bacteria, *Pseudomonas aeruginosa*, twitching motility, type 4 pili

## Abstract

Pseudomonas aeruginosa is among bacterial pathogens capable of twitching motility, a form of surface-associated movement dependent on type IV pili (T4P). Previously, we showed that T4P and twitching were required for P. aeruginosa to cause disease in a murine model of corneal infection, to traverse human corneal epithelial multilayers, and to efficiently exit invaded epithelial cells. Here, we used live wide-field fluorescent imaging combined with quantitative image analysis to explore how twitching contributes to epithelial cell egress. Results using time-lapse imaging of cells infected with wild-type PAO1 showed that cytoplasmic bacteria slowly disseminated throughout the cytosol at a median speed of >0.05 μm s^−1^ while dividing intracellularly. Similar results were obtained with flagellin (*fliC*) and flagellum assembly (*flhA*) mutants, thereby excluding swimming, swarming, and sliding as mechanisms. In contrast, *pilA* mutants (lacking T4P) and *pilT* mutants (twitching motility defective) appeared stationary and accumulated in expanding aggregates during intracellular division. Transmission electron microscopy confirmed that these mutants were not trapped within membrane-bound cytosolic compartments. For the wild type, dissemination in the cytosol was not prevented by the depolymerization of actin filaments using latrunculin A and/or the disruption of microtubules using nocodazole. Together, these findings illustrate a novel form of intracellular bacterial motility differing from previously described mechanisms in being directly driven by bacterial motility appendages (T4P) and not depending on polymerized host actin or microtubules.

## Abstract (precis)

Host cell invasion can contribute to disease pathogenesis by the opportunistic pathogen Pseudomonas aeruginosa. Previously, we showed that the type III secretion system (T3SS) of invasive P. aeruginosa strains modulates cell entry and subsequent escape from vacuolar trafficking to host lysosomes. However, we also showed that mutants lacking either type IV pili (T4P) or T4P-dependent twitching motility (i) were defective in traversing cell multilayers, (ii) caused less pathology *in vivo*, and (iii) had a reduced capacity to exit invaded cells. Here, we report that after vacuolar escape, intracellular P. aeruginosa can use T4P-dependent twitching motility to disseminate throughout the host cell cytoplasm. We further show that this strategy for intracellular dissemination does not depend on flagellin and resists both host actin and host microtubule disruption. This differs from mechanisms used by previously studied pathogens that utilize either host actin or microtubules for intracellular dissemination independently of microbe motility appendages.

## OBSERVATION

Pseudomonas aeruginosa is a leading cause of opportunistic infection at multiple body sites, including the cornea (1, 2). In the cornea and elsewhere, cell invasion and subsequent intracellular survival can promote pathogenesis (3–5). Previously, we demonstrated that cell exit after invasion, the capacity to cross epithelial cell multilayers, and virulence *in vivo* required a type of surface-associated movement called twitching motility (6, 7). Twitching is conferred by type IV pili (T4P), composed of PilA protein, and is accomplished through the extension (dependent on PilB) and retraction (dependent on PilT) of T4P by ATPases that antagonistically polymerize and depolymerize PilA, respectively (8).

Here, we sought to understand how T4P-dependent twitching motility enables P. aeruginosa epithelial cell egress by comparing wild-type invasive P. aeruginosa strain PAO1 to isogenic mutants, namely, a *pilA*::Tn mutant (twitching defective/lacking T4P) and a *pilT*::Tn mutant (twitching defective/possessing T4P) (Table 1) (9). Having previously shown twitching involvement in epithelial cell exit using rabbit corneal epithelial cells (6), we first confirmed the phenotype in human corneal epithelial cells (10). The twitching mutants efficiently invaded these epithelial cells and replicated intracellularly (see Fig. S1A in the supplemental material) but were defective in their capacity for cell egress at 8 h, as previously shown in rabbit cells (6.8-fold lower for the *pilA*::Tn mutant and 10.7-fold lower for the *pilT*::Tn mutant [*P *was ≤0.001 for each versus the wild type, as determined by one-way analysis of variance {ANOVA}]) (Fig. S1B). We also examined HeLa cells. Differing from corneal cells, HeLa cells showed a reduced capacity to internalize a *pilT*::Tn mutant compared to their capacity to internalize the wild type (*P *≤* *0.001, one-way ANOVA) and supported less intracellular replication by the *pilT*::Tn mutant than by the *pilA*::Tn mutant, with 2.6-fold versus 3.8-fold increases, respectively, by 6 h (*P *≤* *0.05, one-way ANOVA comparing numbers of intracellular CFU of the *pilA*::Tn mutant and the *pilT*::Tn mutant) (Fig. S1C). Nevertheless, both twitching mutants were defective in egress from HeLa cells compared to that of wild-type PAO1 (*P *≤* *0.01 for each versus the wild type, by one-way ANOVA) (Fig. S1D). Thus, the role of twitching motility in epithelial cell egress was not specific to corneal epithelial cells.

**TABLE 1:** Strains and plasmids used in this study

Strain or plasmid	Description	Source (reference)
Strains		
mPAO1	Wild type, transposon mutant library parent	PAO1 transposon mutant library (9)
mPAO1 *pilA*::Tn	PW8621 *pilA*-E01::IS*lacZ*/*hah*	PAO1 transposon mutant library (9)
mPAO1 *pilT*::Tn	PW1729 *pilT*-H07::IS*phoA*/*hah*	PAO1 transposon mutant library (9)
mPAO1 *fliC*::Tn	PW8407 *fliC*-B03::IS*phoA*/*hah*	PAO1 transposon mutant library (9)
mPAO1 *flhA*::Tn	PW3636 *flhA*-E11::IS*lacZ*/*hah*	PAO1 transposon mutant library (9)
mPAO1 Δ*pilA*	*pilA* ORF mutant	This study
mPAO1 Δ*pilT*	*pilT* ORF mutant	This study

Plasmids		
pJNE05	T3SS-GFP reporter	Timothy Yahr, University of Iowa (11, 12)
pEXG2	Integrating suicide plasmid	Arne Rietsch, Case Western Reserve University
pMG48	Modified pJNE05 (without the *exoS* promoter)	This study
pMG48*pilA*	*pilA*-GFP dual-function complementation + reporter	This study
pMG48*pilT*	*pilT*-GFP dual-function complementation + reporter	This study

10.1128/mBio.02880-18.2 FIG S1 Human corneal epithelial cells (A, B) and HeLa cells (C, D) were grown as monolayers on tissue culture plates and infected with P. aeruginosa PAO1 or one of its twitching mutants (*pilA*::Tn and *pilT*::Tn mutants) (MOI = 10). Extracellular bacteria were killed with amikacin at 3 h. To quantify intracellular bacteria, cells were lysed at various times after amikacin treatment with 0.25% Triton X-100. Viable-cell counting was performed at 4 h and 8 h postinfection for corneal cells and at 4 h and 6 h for HeLa cells. To quantify bacterial exit, after a 1-h amikacin treatment, fresh medium was placed on the cells and viable-cell counting was performed. (A) PAO1, the *pilA*::Tn mutant, and the *pilT*::Tn mutant showed similar levels of internalization and intracellular survival within corneal epithelial cells (10.7-fold, 28.7-fold, and 20.3-fold increases from 4 h to 8 h, respectively; *P > *0.05). (B) The *pilA*::Tn and *pilT*::Tn mutants showed reduced exit from hTCEpi cells versus that of PAO1 at 8 h (by 6.8-fold and 10.7-fold, respectively; *P *≤* *0.001). (C) In HeLa cells, the *pilA*::Tn mutant showed internalization similar to that of PAO1 at 4 h (*P > *0.05), but its internalization increased 3.8-fold intracellularly by 8 h versus 1.8-fold for PAO1 (*P *≤* *0.05); the *pilT*::Tn mutant showed reduced internalization versus that of PAO1 at 4 h (*P *≤* *0.001), but after 8 h, they reached similar intracellular levels (2.6-fold increase for the mutant; *P > *0.05). The asterisk indicates that the difference in numbers of intracellular CFU between the *pilT*::Tn and *pilA*::Tn mutants at 6 h was significant (*P *≤* *0.05). (D) Both twitching mutants showed reduced exit from HeLa cells at 4 h and 6 h (*P *≤* *0.01). One-way ANOVA with Dunnett’s multiple-comparison test was used for statistical analysis. Download FIG S1, TIF file, 0.2 MB. Copyright © 2019 Nieto et al. 2019 Nieto et al. This content is distributed under the terms of the Creative Commons Attribution 4.0 International license.

**FIG 1:**
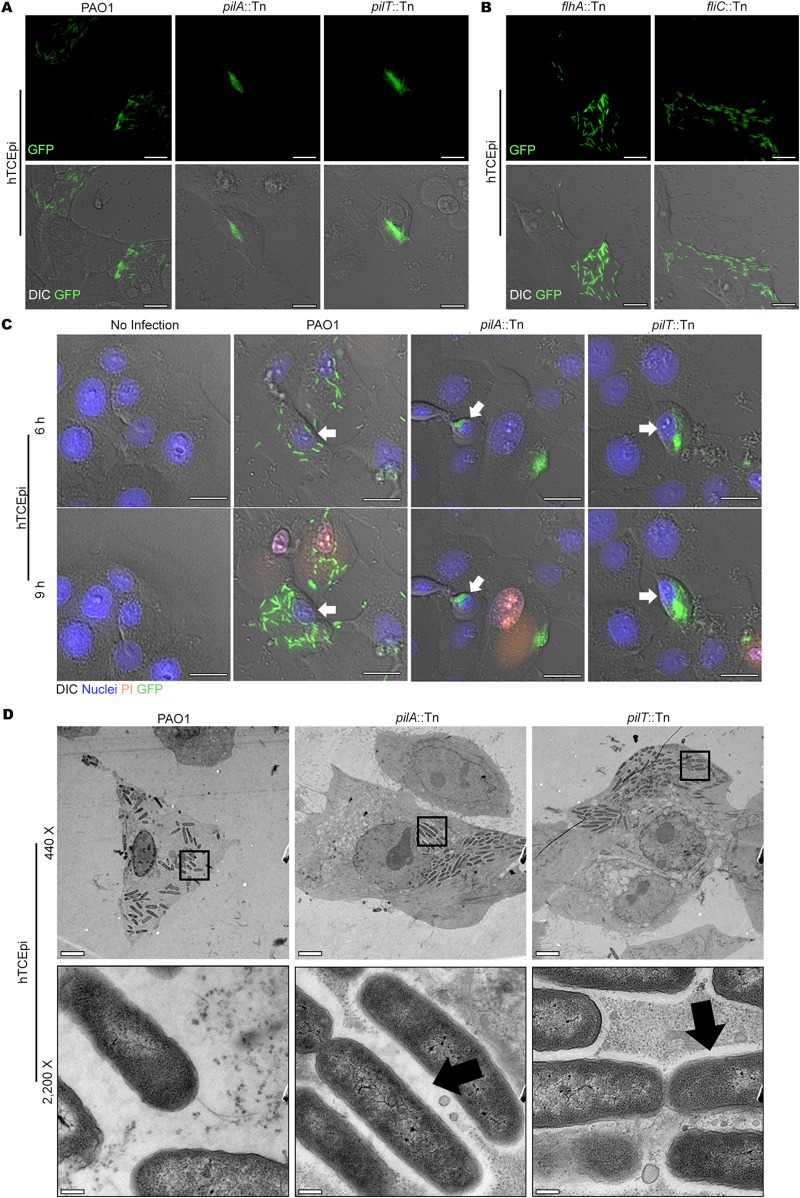
Interactions of P. aeruginosa PAO1 and its twitching (*pilA*::Tn or *pilT*::Tn), swimming (*flhA*::Tn; flagellum rod), and *fliC*::Tn (flagellin) motility mutants harboring the T3SS reporter pJNE05 (GFP) with human corneal epithelial cells (hTCEpi) (multiplicity of infection [MOI] = 10). (A) Time-lapse video microscopy images (7 h postinfection) show T3SS-expressing PAO1 dispersed intracellularly, while T3SS-positive twitching mutants form intracellular aggregates. Bars = 20 μm. (B) Time-lapse video microscopy images of intracellular T3SS-expressing PAO1 swimming mutants (*flhA*::Tn and *fliC*::Tn mutants) at 7 h postinfection showing intracellular dispersal. Bars = 20 μm. (C) Propidium iodide (PI) permeability of human corneal epithelial cell monolayers after P. aeruginosa exposure. Cells were infected with P. aeruginosa PAO1 or its twitching mutants (the *pilA*::Tn or *pilT*::Tn mutant) harboring the T3SS-GFP reporter plasmid (pJNE05) (MOI = 10). Extracellular bacteria were killed with amikacin at 3 h postinfection, and cells were imaged from 4 h using time-lapse video microscopy; 6 h and 9 h postinfection are shown. Arrows point to living corneal cells containing bacteria (PI impermeable, no staining) at 6 h during bacterial replication, dispersal of T3SS-positive intracellular PAO1, and the formation of intracellular aggregates by twitching mutants. After 9 h, more corneal epithelial cells labeled with PI, as expected, but viable cells containing intracellular bacteria remained (white arrows). Bars = 20 μm. (D) TEM of infected corneal cells at 6 h (extracellular bacteria killed with amikacin at 3 h) showing PAO1 dispersed throughout the cytoplasm and twitching mutants as intracellular aggregates. The cytoplasm of PAO1-infected cells was more electron lucent than that of twitching mutants. At magnifications of ×440 and ×2,200 (the boxed areas in the ×440 images), the *pilA*::Tn and *pilT*::Tn mutants exhibited conjoined electron-lucent halos (black arrows) in the majority of individual infected cells that were not apparent after PAO1 infection. Bars = 5 μm (magnification, ×440) and 0.2 μm (magnification, ×2,200).

Next, we used imaging to compare twitching mutants to the wild type. A type III secretion system-green fluorescent protein (T3SS-GFP) reporter was used since we have previously shown that it provides a reliable marker for imaging intracellular P. aeruginosa (11, 12). Bacteria within the cell cytoplasm appeared stationary in real time for both the wild type and twitching mutants. However, time-lapse imaging showed wild-type bacteria slowly disseminating throughout the cytoplasm in a pattern reminiscent of twitching motility, while rapidly replicating intracellularly (Fig. 1A; Movies S1 and S2). Both twitching mutants (i.e., with and without T4P) remained stationary and instead formed intracellular aggregates that expanded in size during intracellular division (Fig. 1A; Movie S2). The T3SS reporter confirmed that twitching mutants also showed T3SS expression when internalized (Fig. 1A; Movie S2), as previously reported for the wild type (11, 12). These twitching mutant phenotypes (intracellular aggregation, T3SS expression) were also verified using transposon-free clean deletion mutants devoid of *pilA* or *pilT* open reading frames (ORFs) (Movie S3, upper panels). Complementation of these mutants in *trans* with cloned *pilA* or *pilT* constructs (Table 1 and Table 2) restored intracellular motility during corneal cell infection (Movie S3, lower panels). An intracellular-aggregation phenotype of *pilA* and *pilT* mutants was also observed in infected HeLa cells (Fig. S2). Thus, T4P-dependent twitching motility was found to be required for intracytoplasmic motility by wild-type PAO1 for multiple epithelial cell types.

**TABLE 2:** Primers used for mutagenesis or molecular cloning

Primer name	Sequence^*a*^
*pilA* pEXG2 Gibson F	5′-ggaagcataaatgtaaagcaGCTTTCGAACAGCTTGTCGATGG-3′
*pilA* pEXG2 Gibson R	5′-ggaaattaattaaggtaccgGTCACCTGCGGCGGTTGC-3′
*pilA* pEXG2 deletion F	5′-CTACCCAGGATCCGATGT-3′
*pilA* pEXG2 deletion R	5′-CAGTTCGATCAAGGTAAAGC-3′
*pilT* pEXG2 Gibson F	5′-ggaagcataaatgtaaagcaACTGGAAATGCTCGGCGATG-3′
*pilT* pEXG2 Gibson R	5′-ggaaattaattaaggtaccgAGCGAGGTGGACTTGCCG-3′
*pilT* pEXG2 deletion F	5′-CTCGCTGGGCATGCAGAC-3′
*pilT* pEXG2 deletion R	5′-GGTTGATCCGGCGTACATC-3′
*pilA* pMG48 Gibson F	5′-gttagttagggaataagccgCCTTCGATCACCTTAGTTATCAC-3′
*pilA* pMG48 Gibson R	5′-taccggaattggggatcggaGGGGAAGGAATCGCAGAAG-3′
*pilT* pMG48 Gibson F	5′-gttagttagggaataagccgGGATCGGCGCCAGGATCA-3′
*pilT* pMG48 Gibson R	5′-taccggaattggggatcggaTACCTGCGCCCTATGGAAG-3′

a Lowercase letters indicate the segment of primer that anneals to the vector. Uppercase letters indicate the segment of primer that anneals to the PAO1 genome. All primers were generated by this study.

10.1128/mBio.02880-18.3 FIG S2 HeLa cells were infected with P. aeruginosa PAO1 or its twitching mutants (*pilA*::Tn or *pilT*::Tn mutant) harboring the T3SS-GFP reporter plasmid (pJNE05) (MOI = 10). Cells were imaged using time-lapse video microscopy (the result at 7 h postinfection is shown). T3SS-positive intracellular PAO1 cells dispersed within the cytosol. In contrast, T3SS-positive twitching mutants formed intracellular aggregates. Bars = 20 μm. Download FIG S2, TIF file, 1.5 MB. Copyright © 2019 Nieto et al. 2019 Nieto et al. This content is distributed under the terms of the Creative Commons Attribution 4.0 International license.

10.1128/mBio.02880-18.5 MOVIE S1 Combined wide-field DIC microscopy with fluorescence time-lapse imaging of intracellular P. aeruginosa PAO1 expressing the T3SS-GFP reporter (pJNE05) moving within the cytoplasm of human corneal epithelial cells from 4 to 10 h postinoculation. Extracellular amikacin was present from 3 h postinoculation (×40 magnification, 1 frame captured every 15 min, video rendered at 4 frames per second [FPS]). Download Movie S1, MOV file, 9.1 MB. Copyright © 2019 Nieto et al. 2019 Nieto et al. This content is distributed under the terms of the Creative Commons Attribution 4.0 International license.

10.1128/mBio.02880-18.6 MOVIE S2 Combined wide-field DIC microscopy with fluorescence time-lapse imaging of intracellular P. aeruginosa PAO1 expressing the T3SS-GFP reporter (pJNE05) moving within the cytoplasm of human corneal epithelial cells from 4 to 10 h postinoculation. Twitching motility mutants, i.e., the *pilA*::Tn and *pilT*::Tn mutants, also showed T3SS activation but formed intracellular aggregates. Extracellular amikacin was present from 3 h postinoculation (×40 magnification, 1 frame captured every 15 min, video rendered at 4 FPS). Download Movie S2, MOV file, 8.4 MB. Copyright © 2019 Nieto et al. 2019 Nieto et al. This content is distributed under the terms of the Creative Commons Attribution 4.0 International license.

10.1128/mBio.02880-18.7 MOVIE S3 (Top) Combined wide-field DIC micrographs with fluorescence time-lapse imaging of intracellular P. aeruginosa PAO1 cells expressing the T3SS-GFP reporter (pJNE05) moving in the cytoplasm of human corneal epithelial cells from 4 to 10 h postinoculation. Transposon-less clean-deletion twitching motility mutants, the Δ*pilA* and Δ*pilT* mutants, also showed T3SS activation but formed intracellular aggregates (membrane blebs were also evident, but without bacteria in this instance). (Bottom) Combined wide-field DIC micrographs with fluorescence time-lapse imaging of PAO1 (left) and the clean-deletion twitching motility Δ*pilA* and Δ*pilT* mutants, expressing the complementation vectors pMG48*pilA* (p*pilA*) and pMG48*pilT* (p*pilT*), respectively (see Text S1 and Table 1), in human corneal epithelial cells from 4 to 10 h postinoculation. Complementation with *pilA* or *pilT* in *trans* restored intracellular motility similar to that of the wild type. Note that the GFP expression in the lower middle and right panels reflects the expression of *pilA* or *pilT* rather than of the T3SS. Extracellular amikacin was present from 3 h postinoculation (×40 magnification, 1 frame captured every 15 min, video rendered at 4 FPS). Download Movie S3, MOV file, 9.1 MB. Copyright © 2019 Nieto et al. 2019 Nieto et al. This content is distributed under the terms of the Creative Commons Attribution 4.0 International license.

10.1128/mBio.02880-18.1 TEXT S1 Supplemental materials and methods. Download Text S1, DOCX file, 0.03 MB. Copyright © 2019 Nieto et al. 2019 Nieto et al. This content is distributed under the terms of the Creative Commons Attribution 4.0 International license.

Previously, we showed that wild-type PAO1 can use its T3SS to form membrane blebs in epithelial cells to which a fraction of intracellular bacteria traffic (11–13), with even greater bleb formation, bacterial occupation, and intracellular replication found in epithelial cells from a patient with cystic fibrosis (14). Indeed, in the experiments described above, membrane bleb formation was observed in human corneal epithelial cells infected with a *pilA* or *pilT* mutant, although those particular blebs did not contain bacteria (Movie S3, upper panels). When P. aeruginosa occupies these “bleb niches,” which are devoid of cytoskeletal structures and which can disconnect from the epithelial cell, it demonstrates swimming motility detectable by real-time observation (11, 13). Thus, we explored whether swimming might synergize with twitching for motility in the cytoplasm. Since P. aeruginosa swimming depends on a single polar flagellum (15), we used a flagellum assembly mutant (*flhA*::Tn mutant) and a flagellin mutant (*fliC*::Tn mutant) (15) after confirming that they could activate the T3SS intracellularly (Fig. 1B; Movie S4). As observed for wild-type bacteria, both swimming mutants disseminated within infected cells (Fig. 1B; Movie S4). This suggested that swimming motility was not involved in cytoplasmic dissemination, and neither could swarm or slide (the former requiring both flagella and T4P function, the latter depending on their combined absence) (16, 17).

10.1128/mBio.02880-18.8 MOVIE S4 Combined wide-field DIC micrographs with fluorescence time-lapse imaging of swimming mutants of P. aeruginosa, namely, the *flhA*::Tn mutant (flagellum biosynthesis mutant) and *fliC*::Tn mutant (flagellin mutant) expressing the T3SS-GFP reporter (pJNE05) and moving within human corneal epithelial cells from 4 to 10 h postinoculation. Extracellular amikacin was present from 3 h postinoculation (×40 magnification, 1 frame captured every 15 min, video rendered at 4 FPS). Download Movie S4, MOV file, 8.6 MB. Copyright © 2019 Nieto et al. 2019 Nieto et al. This content is distributed under the terms of the Creative Commons Attribution 4.0 International license.

Propidium iodide (PI) was used to visualize dead or dying human corneal epithelial cells during P. aeruginosa exposure to determine if host cells containing intracellular bacteria were viable. After 6 h, the majority of host cells remained viable, and intracellular bacteria (motile wild type, nonmotile *pilA* and *pilT* mutants) were observed inside viable cells, i.e., in the absence of PI labeling (Fig. 1C, white arrows). While some dead or dying (PI-labeled) host cells were observed after 9 h, including those containing bacteria, other host cells containing intracellular bacteria remained viable (no PI labeling) despite significant bacterial replication and intracellular motility (Fig. 1C, white arrows).

A potential mechanism for intracellular aggregation of twitching mutants is if the mutants are trapped inside a membrane-bound vacuole. Such is the fate of T3SS mutants, unable to escape endocytic trafficking once internalized by an epithelial cell (12, 13, 18). Thus, we used transmission electron microscopy (TEM) to study bacterial location within infected cells. Results showed neither the wild type nor twitching mutants surrounded by membranous material intracellularly (Fig. 1D), showing that they had escaped vacuoles and were in the host cell cytoplasm. However, the cytoplasm of wild-type-infected cells was more electron lucent (78% of individual cells [*n* = 23]) than that of cells infected with either twitching mutant (31.6% and 27.8% of cells for the *pilA*::Tn mutant [*n* = 19] and the *pilT*::Tn mutant [*n* = 18], respectively) (*P* < 0.01, Fisher’s exact test). This suggested a differential expression of T3SS effectors, known to be capable of disrupting the actin cytoskeleton (19, 20). However, both wild-type- and mutant-infected cells were rounded, a phenomenon known to depend on T3SS effectors.

Possibly relevant, intracytoplasmic twitching mutants were surrounded by conjoined electron-lucent halos (black arrows) in 87% (*n* = 19) and 88.9% (*n* = 18) of cells infected with the *pilA*::Tn and *pilT*::Tn mutants, respectively, apparent in only 13% (*n* = 23) of wild-type PAO1 cells (*P* < 0.0001, Fisher’s exact test). Why this occurs will require further investigation. Hypotheses include that wild-type intracellular motility might help spread secreted T3SS effectors throughout the cytosol to produce a more generalized cytoskeletal disruption. Also possible is that twitching mutants form intracellular biofilms (21, 22), with electron-lucent silhouetting representing extracellular products (e.g., exopolysaccharide or extracellular DNA), which may also relate to reduced egress of these mutants.

**FIG 2:**
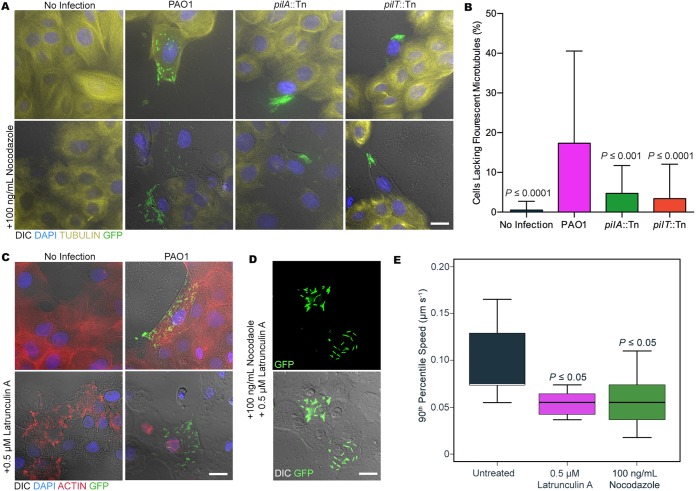
(A) Human corneal epithelial cells (hTCEpi) were infected with P. aeruginosa PAO1 or its twitching mutants (the *pilA*::Tn and *pilT*::Tn mutants), each containing the T3SS-GFP reporter plasmid pJNE05 (MOI = 10). Some infected cells were treated with 100 ng/ml nocodazole at 3 h postinoculation along with amikacin to kill extracellular bacteria (see Text S1 in the supplemental material). Immunofluorescence images after 6 h show that wild-type and twitching mutants expressed the T3SS but that nocodazole treatment (lower panels) did not visibly affect the intracellular motility of PAO1 or the intracellular aggregation of the twitching mutants. (B) Quantification of fluorescent microtubules (labeled with antibody versus β-tubulin) in P. aeruginosa-infected hTCEpi cells (prepared as described for panel A) was performed by randomly acquiring visual fields (*n* = 37) and manually counting cells. PAO1-infected corneal cells exhibited a greater mean loss of fluorescent microtubules (17.4%) than uninfected cells (0.52%) or cells infected with the *pilA*::Tn mutant (4.7%) or the *pilT*::Tn mutant (3.4%) (*P *≤* *0.0001, *P *≤* *0.001, or *P *≤* *0.0001, respectively, by one-way ANOVA and Dunnett’s multiple-comparison test). (C) hTCEpi cells were infected with P. aeruginosa strain PAO1 containing the T3SS-GFP reporter as described above with and without 0.5 μM latrunculin A added at 3 h postinoculation. Immunofluorescence images at 6 h postinoculation show that latrunculin A did not appear to affect PAO1 intracellular motility (lower panels). (D) At 6 h postinoculation, PAO1 intracellular motility was also unaffected when hTCEpi cells were infected and treated with both 100 ng/ml nocodazole and 0.5 μM latrunculin A (added at 3 h postinoculation). (E) The velocity of intracellular bacteria expressing the T3SS reporter was measured computationally using time-lapse imaging of cells infected with PAO1 *fliC*::Tn with and without 0.5 μM latrunculin A or 100 ng/ml nocodazole after 6 h of infection. The PAO1 *fliC*::Tn median twitching speed was 0.074 μm s^−1^, significantly higher than in cells treated with nocodazole or latrunculin A, both of which were measured at 0.055 μm s^−1^ (*P = *0.011 and 0.028, respectively, for each versus the control [one-sided Wilcoxon test]). There was no significant difference in intracellular bacterial velocities between nocodazole- and latrunculin A-treated cells (*P > *0.05, one-sided Wilcoxon test). Bars = 20 μm. DIC, differential inference contrast; DAPI, 4′,6-diamidino-2-phenylindole.

Other bacterial pathogens manipulate host cell cytoskeletal components, either microtubules or actin, for motility in the host cell cytoplasm (3, 23, 24). Thus, we studied the impact of nocodazole, an agent that depolymerizes microtubules (25). Human corneal epithelial cells were inoculated with PAO1 or its twitching mutants as described in the legend of Fig. 1 and incubated them for 3 h, at which point nocodazole (100 ng/ml) was added for another 3 h along with amikacin to kill extracellular bacteria. After 6 h, infected cells were examined by time-lapse imaging and immunofluorescence microscopy (Fig. 2). Controls confirmed that nocodazole had disrupted microtubule structure in the experiments (Fig. 2A) but had no impact on the intracellular dissemination of wild-type PAO1 (Fig. 2A; Movie S5). Nocodazole treatment also had no visible impact on the intracellular aggregation of either twitching mutant (Fig. 2A).

10.1128/mBio.02880-18.9 MOVIE S5 Combined wide-field DIC micrographs with fluorescence time-lapse imaging of P. aeruginosa PAO1 expressing the T3SS-GFP reporter (pJNE05) and moving within the cytosol of human corneal epithelial cells (×40 magnification, 1 frame captured every 10 min, video rendered at 4 FPS). PAO1 (dimethyl sulfoxide [DMSO] only) was used as a control. At 3 h postinoculation, cells were treated with 100 ng/ml nocodazole, 0.5 μM latrunculin A, or a combination of both at the same concentrations, which continued throughout the assay. The video shows 4 to 10 h postinoculation. Extracellular amikacin was also present from 3 h postinoculation onwards. Download Movie S5, MOV file, 12.4 MB. Copyright © 2019 Nieto et al. 2019 Nieto et al. This content is distributed under the terms of the Creative Commons Attribution 4.0 International license.

Microtubule structure and location relative to those of intracellular P. aeruginosa (T3SS-expressing, green) were examined by labeling microtubules with antibody against β-tubulin (yellow). Instead of aligning with microtubules, intracellular P. aeruginosa disrupted microtubule filaments in both infected cells and adjacent cells (Fig. 2A). Relevant here, PAO1 expresses the T3SS when it is intracellular (11), and it encodes the effector ExoY, which can disrupt microtubules via hyperphosphorylation of tau (19, 26). Shigella flexneri is another pathogen capable of intracytoplasmic motility that can degrade microtubules during infection (27). Both twitching-defective mutants also triggered T3SS expression intracellularly and impacted microtubule structure (Fig. 2A, upper panels), but their impact was greatly reduced compared to that of wild-type PAO1 (Fig. 2B), which may relate to differences in electron lucidity within the infected cell cytoplasm noted previously.

While these results suggest that P. aeruginosa does not depend on microtubules for its intracellular motility, it is possible that microtubule degradation can modulate the intracellular behavior of twitching-competent wild-type P. aeruginosa. Changes to cytoskeleton components, such as microtubules or intermediate filaments, can modulate trafficking of other bacteria within the cytoplasm of host cells (28, 29).

Various bacterial pathogens (e.g., S. flexneri and Listeria monocytogenes) utilize host cell actin to enable their intracellular motility (24). Time-lapse movies of P. aeruginosa intracellular trafficking showed linear movement not resembling the trajectory curvature of typical actin polymerization that drives intracytoplasmic motility by other bacteria. In case actin played nonclassical roles, we explored the impact of the actin-depolymerizing agent latrunculin A (30). Human corneal epithelial cells were treated with 0.5 μM latrunculin A at the times and conditions described above for nocodazole. Actin filaments were disrupted by latrunculin A in these cells but had no visible impact on P. aeruginosa intracellular motility (Fig. 2C; Fig. S3; Movie S5).

10.1128/mBio.02880-18.4 FIG S3 Human corneal epithelial cells (hTCEpi) were infected with P. aeruginosa PAO1 containing the T3SS-GFP reporter plasmid pJNE05 (MOI = 10). Some infected cells were treated with 0.5 μM latrunculin A, added at 3 h postinoculation along with amikacin to kill extracellular bacteria (Text S1). The actin cytoskeleton was labeled with Alexa Fluor 555-phalloidin. Immunofluorescence images captured at 6 h postinoculation show that latrunculin A did not appear to affect PAO1’s intracellular motility despite disruption of its actin cytoskeleton. Download FIG S3, TIF file, 1.9 MB. Copyright © 2019 Nieto et al. 2019 Nieto et al. This content is distributed under the terms of the Creative Commons Attribution 4.0 International license.

The combined use of nocodazole and latrunculin A to disrupt both microtubules and actin filaments, respectively, in the same cells also had no obvious impact on the intracellular dissemination of wild-type P. aeruginosa (Fig. 2D; Movie S5), nor did they visibly impact intracellular aggregation of twitching mutants (data not shown).

To further explore the relationship between P. aeruginosa intracellular motility and classical T4P-dependent twitching motility, computational analysis was used to study intracellular velocity. To better focus on T4P-dependent intracellular motility and avoid bacteria swimming within membrane blebs (11, 13), we used flagellin (PAO1 *fliC*::Tn) mutants, which are competent for T4P-dependent intracellular motility. Since intracellular bacteria followed common paths and formed clusters within cells, the velocity of bacterial motility was quantified by measuring the moment of displacement of each bacterium between pairs of acquired frames, allowing generation of a distribution of moment velocities of individual bacteria (see Text S1 in the supplemental material). The PAO1 wild type exhibited a median velocity of 0.074 μm s^−1^ (Fig. 2E; Movie S6) in cells, with similar results obtained with and without nocodazole and/or latrunculin A treatment. These results closely matched published values for P. aeruginosa twitching motility on *in vitro* surfaces (7, 31, 32).

10.1128/mBio.02880-18.10 MOVIE S6 Wide-field fluorescence time-lapse imaging of P. aeruginosa PAO1 *fliC*::Tn (flagellin mutant) and PAO1 expressing the T3SS-GFP reporter (pJNE05) moving within human corneal epithelial cells at 6 h postinfection (×40 magnification, 1 frame per 10 s, 5 min total). Time-lapse images were processed using ImageJ computational analysis to form a red-green-blue (RGB) velocity map of bacterial intracellular motility. (Left) Control (DMSO); (middle) 0.5 μM latrunculin A; (right) 100 ng/ml nocodazole. Cytoskeleton inhibitors did not prevent intracellular motility. Download Movie S6, MOV file, 1.6 MB. Copyright © 2019 Nieto et al. 2019 Nieto et al. This content is distributed under the terms of the Creative Commons Attribution 4.0 International license.

Surprisingly, disruption of either microtubules or actin resulted in somewhat lower median twitching velocities, differences that were statistically significant, although values were still >0.05 μm s^−1^ (Fig. 2E). Controls confirmed that neither of the inhibitors affected bacterial viability. Thus, while polymerized actin and/or microtubules are not required for P. aeruginosa to disseminate in the cytoplasm, both can influence the process beyond forming barriers that prevent movement, which would have produced the opposite result.

T4P have been shown to be required for T3SS (ExoU)-mediated cytotoxicity by asialo-GM1 binding; the T3SS also facilitates the internalization of T3SS-null P. aeruginosa (33). T4P can also function as mechanotransducers activating the Chp chemosensory system and, hence, multiple virulence determinants, including Vfr, a positive regulator of the T3SS (34). The present study suggests that the relationship between T4P and the T3SS may be less clear for intracellular P. aeruginosa since both the *pilA* and *pilT* mutants showed T3SS-GFP reporter expression similar to that of the wild type. Moreover, the absence of vacuolar membranes around intracellular *pilA* and *pilT* mutants, induction of membrane blebs, and epithelial cell rounding all suggest T3SS (ExoS) expression (11–14). If so, this may be a promising avenue of further investigation. For example, is there any relationship to our previous observation that corneal epithelial cell lysates can induce ExoS expression (35)?

In summary, this study shows that intracellular dissemination of P. aeruginosa throughout the cytoplasm of epithelial cells depends on T4P and twitching motility. Mutants lacking twitching remain localized in cytosolic aggregates, while still triggering T3SS expression and not being bound by host membrane material. Although cytoskeletal elements had a minor impact on bacterial speed, they were not required for cytoplasmic dissemination. In fact, microtubules were disrupted even more efficiently by P. aeruginosa competent for twitching-dependent intracellular motility.

The pattern, speed, and other characteristics of P. aeruginosa motility in the cytoplasm of epithelial cells, including relative independence from host actin and microtubules, suggest that motility is driven primarily by T4P twitching function, akin to how pili move along abiotic surfaces. This differs from previously described bacterial intracellular motility mechanisms that are driven primarily by host cytoskeletal components independently of bacterial motility appendages. How the role of twitching motility in cytoplasmic dissemination relates to its previously established contribution to host cell exit remains to be determined.
