# Supplementary figures and images for: Type IV Pili Can Mediate Bacterial Motility within Epithelial Cells
Source: mBio. 2019 Aug 20;10(4):e02880-18. doi: 10.1128/mBio.02880-18 (PMC6703432; doi:10.1128/mBio.02880-18)

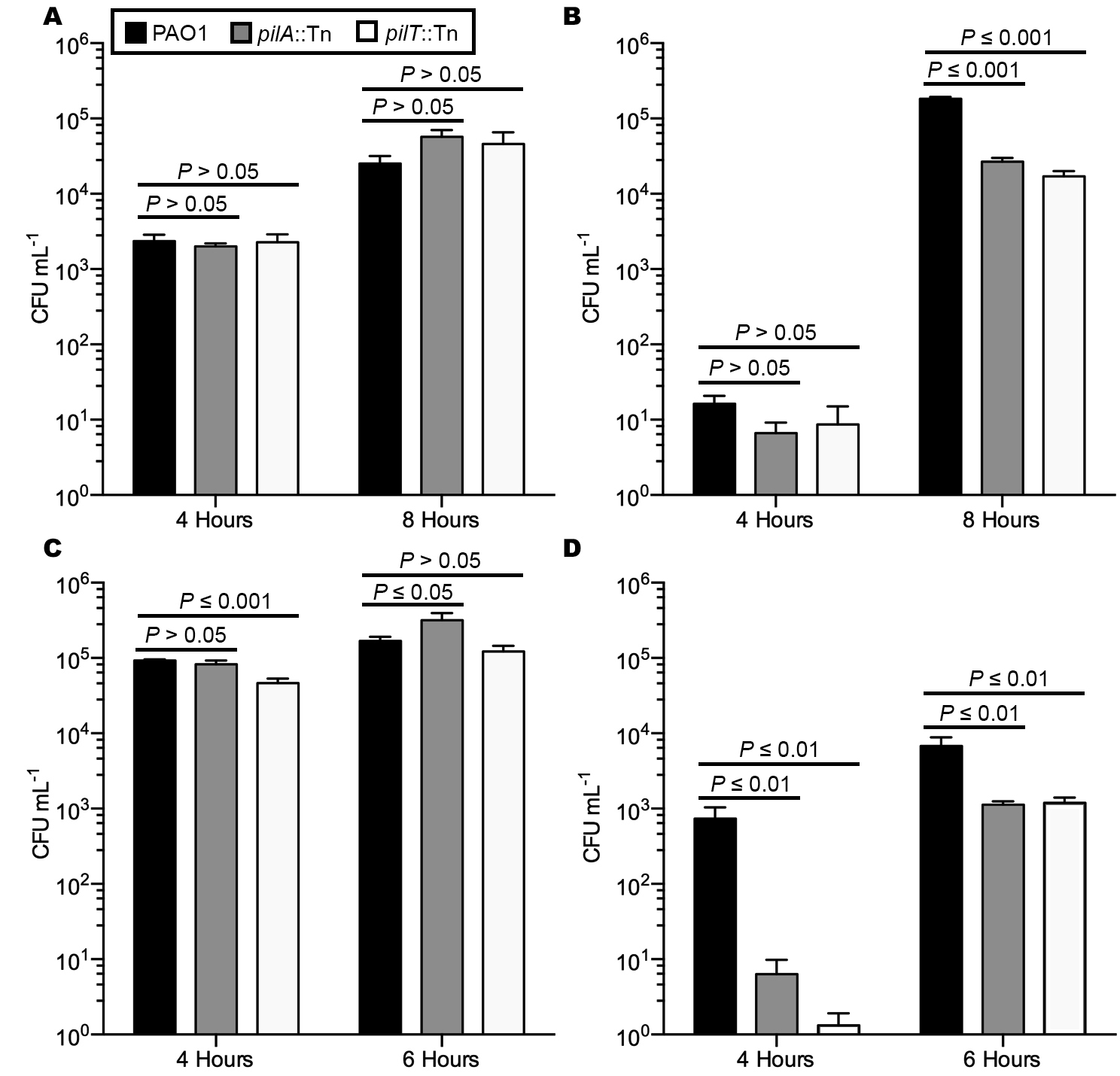

Supplement: FIG S1 [file mBio.02880-18-sf001.tif]

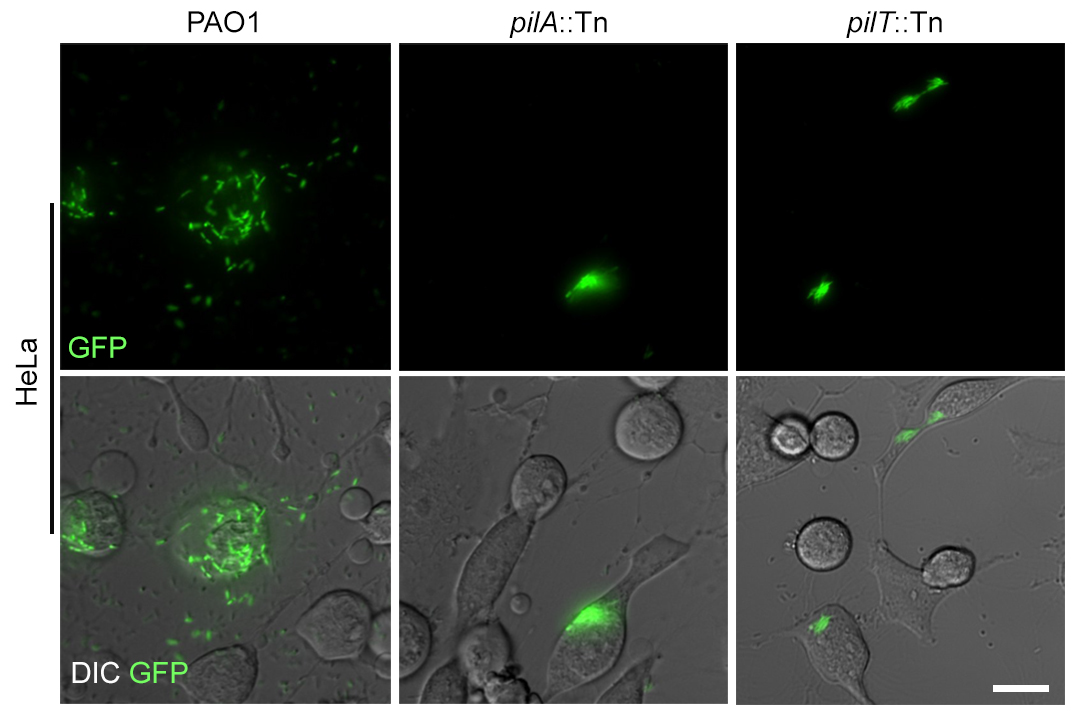

Supplement: FIG S2 [file mBio.02880-18-sf002.tif]

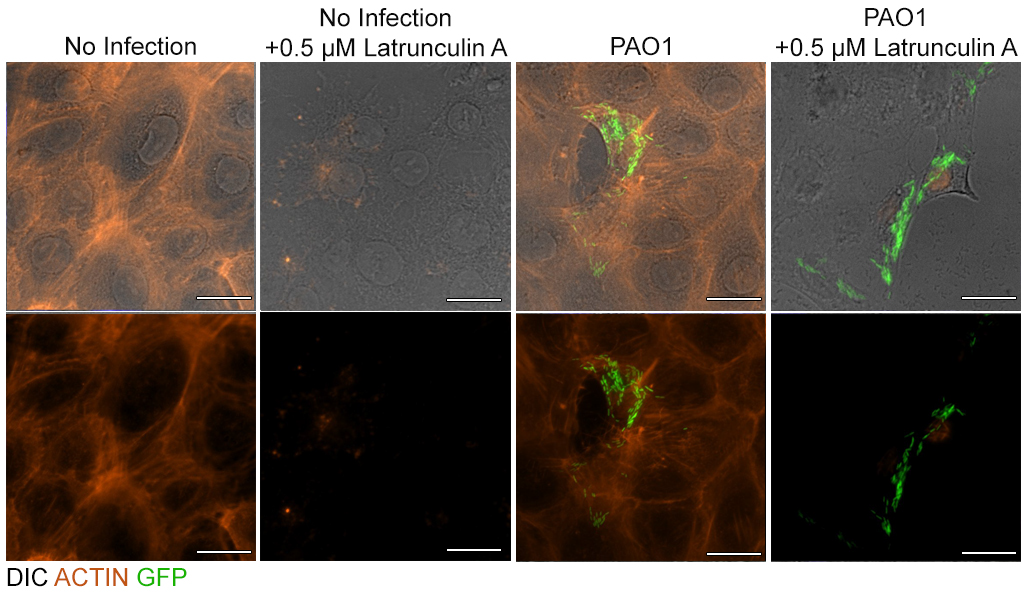

Supplement: FIG S3 [file mBio.02880-18-sf003.tif]
