# Supplementary material for: Type IV Pili Can Mediate Bacterial Motility within Epithelial Cells
Source: mBio. 2019 Aug 20;10(4):e02880-18. doi: 10.1128/mBio.02880-18 (PMC6703432; doi:10.1128/mBio.02880-18)
Supplement: TEXT S1 [file mBio.02880-18-s0001.docx]

**Materials and Methods**

Bacterial strains, plasmids, and mutants

*P. aeruginosa* strains and plasmids used are shown in Table 1. *P. aeruginosa* strain PAO1, its twitching motility mutants: *pilA*::Tn (pilin) and *pilT*::Tn (pilus-retraction), and its swimming mutants: *flhA*::Tn (flagellum assembly) and *fliC*::Tn (flagellin subunit) were obtained from the *P. aeruginosa* PAO1 transposon mutant library (Dr. Manoil Laboratory, University of Washington, Seattle, WA) (7). Bacteria were grown at 37 °C on Trypticase Soy Agar (TSA) (Hardy Diagnostics, Santa Maria, CA) or TSA + tetracycline (10 µg/mL) for 16 h prior to use. For imaging, bacteria were transformed a type three secretion system (T3SS)-GFP reporter plasmid (pJNE05) (11, 12), and grown at 37 ^o^C on TSA supplemented with gentamicin (200 µg/mL). The *pilA*::Tn mutant and both *flhA*::Tn and *fliC*::Tn mutants were previously verified (7, 11). Clean deletions of *pilA* and *pilT* genes in PAO1 were engineered by allelic exchange as follows: 500-bp regions flanking *pilA* or *pilT* were amplified (see Table 2 for primers) using Q5^®^ High-Fidelity DNA Polymerase (New England Biolabs, Ipswich, MA). Fragments were cloned into pEXG2 using EcoR1 and HindIII restriction sites and the Gibson Assembly Cloning Kit (New England Biolabs). An open reading frame (ORF) mutant of *pilA* or *pilT* was created by using internal primers to *pilA* or *pilT* clones (see Table 2) and with the Q5^®^ Site-Directed Mutagenesis Kit (New England Biolabs), leaving the start and stop codon of *pilA* and *pilT* intact. *P. aeruginosa* was transformed by conjugation with *Escherichia coli* SM10 on LB agar and counter-selected on 5% sucrose low-salt LB agar. Mutants were confirmed by PCR, sequencing and motility tested using microscopy of strains grown on gellan gum twitching media, as previously described (7). Complementation of *pilA* or *pilT* was performed as followed: 200-bp upstream of each *pilA* or *pilT* gene was amplified along with the respective gene ORF (see Table 2) using Q5^®^ High-Fidelity DNA Polymerase (New England Biolabs). Fragments were cloned using EcoR1 and HindIII restriction sites and the Gibson Assembly Cloning Kit (New England Biolabs) into a modified pJNE05 (11, 12) vector, pMG48, lacking the *exoS* (T3SS) promoter, but retaining GFP. The placement of the *pilA* or *pilT* fragments drove the expression of downstream GFP, creating a dual-function complementation and reporter vector for *pilA* (pMG48*pilA*) or *pilT* (pMG48*pilT*) (Table 1). Gene complementation was verified through microscopic analysis using gellan gum twitching media (7) and intracellular motility visualized by time-lapse fluorescence microscopy.

Cell culture

Human corneal epithelial cells (hTCEpi) were cultured in KGM-2 (Lonza, USA) supplemented with 1.15 mM calcium (high-calcium) for differentiation and maintained at 37 °C (humidified) in a 5 % CO_2_ (vol./vol.) incubator. HeLa cells were cultured in DMEM (Thermo Fisher Scientific, Waltham, MA) with 10 % fetal bovine serum (FBS) (ThermoFisher) and incubated as for hTCEpi.

Bacterial internalization, intracellular replication and exit assays

Human corneal epithelial cells and HeLa cells were grown as monolayers on 96-well tissue culture plates. Bacteria inocula were prepared by resuspending bacterial colonies in sterile PBS to an optical density at 550 nm of 1.0 (~ 2 x 10^8^ CFU/mL). Cells were inoculated with 5 µL of suspension (MOI = 10) of PAO1 or twitching motility mutants (*pilA*::Tn, or *pilT*::Tn) containing a T3SS-GFP reporter, and incubated for 3 h at 37 °C (internalization period). Cells were then incubated with cell culture medium (KGM-2 + 1.15 mM calcium, or DMEM + 10% FBS) supplemented with 200 μg/mL amikacin (Sigma-Aldrich, St. Louis, MO) for 1 h to kill extracellular bacteria. Bacterial exit from cells after internalization and intracellular replication was quantified by removing amikacin-containing medium from the cells at 1 h after the internalization period, adding fresh KGM-2 (with 1.15 mM calcium without antibiotics) for 1 h (exit period). Viable counts of that extracellular medium allowed enumeration of viable bacteria that had exited cells during that hour. Additional samples were included to enumerate intracellular bacteria at various times post-inoculation. In those samples, cells were treated with amikacin-containing media for at least 1 h to kill extracellular bacteria, followed by 15 min treatment with 0.25 % (vol./vol.) triton X-100 (Sigma-Aldrich) in PBS to lyse the cells, then viable counts on aliquots of the cell lysate.

Wide-field imaging

Live and time-lapse images were captured on a Nikon Ti-E inverted wide-field fluorescence microscope equipped with Lumencor SpectraX illumination source and Okolab Uno-combined controller stage top incubation chamber to maintain heat, humidity, and 5% CO_2_. Time-lapse images were captured using a CFI Plan Apo Lambda 40X air objective, equipped with differential interference contrast (DIC). For time-lapse, fields were chosen visualizing DIC only to identify areas free of debris; GFP was not observed until time-lapse was completed to avoid bias in field selection. For time-lapse, four fields were imaged for each condition.

Propidium iodide labeling

Propidium iodide (PI) (ImmunoChemistry Technologies, Bloomington, MN) was used label dead or dying host cells. Specifically, live human corneal epithelial cells were cultured in the presence of PI and counterstained with NucBlue® Live ReadyProbes® Reagent (Thermo Fisher Scientific) to label all cell nuclei. At various time points after bacterial inoculation, cells were imaged using wide-field imaging (above).

Immunofluorescence

Human corneal epithelial cells were grown as above but on glass coverslips with KGM-2 (+ 1.15 mM calcium) then inoculated with *P. aeruginosa* PAO1, *pilA*::Tn*,* or *pilT*::Tn containing the T3SS-GFP reporter at a MOI of 10, and incubated for 3 h at 37 °C. Fresh KGM-2 (+ 1.15 mM calcium) containing amikacin (200 μg/mL) was then added to kill extracellular bacteria for 3 h at 37 °C. At 6 h post-inoculation, cells were washed with PBS, and fixed in fresh 4 % paraformaldehyde (PFA) (Sigma-Aldrich) for 10 min. After an additional PBS wash, cells were permeabilized for 10 min using 4 % PFA containing 0.1 % triton X-100. Cells were quenched with aldehydes in 150 mM glycine (Sigma-Aldrich) for 10 min, washed in PBS, and blocked for 1 h at room temperature in 2.5 % fish skin gelatin (Sigma-Aldrich), 5 % FBS, 0.1 % triton X-100, 0.05 % tween 20 (Sigma-Aldrich). For Microtubules: primary rabbit monoclonal antibody (mAb) targeting β-tubulin (Cell Signaling Technology, Danvers, MA) in 1.25 % fish skin gelatin, 2.5 % FBS, 0.5 % triton X-100, 0.25 % tween 20 was applied to cells for 16 h. Cells were washed with PBS and secondary goat anti-rabbit IgG (H+L) antibody, AlexaFluor 555 (Thermo Fisher Scientific) was applied in 1.25 % fish skin gelatin, 2.5 % FBS, 0.5 % tritonX-100, 0.25 % tween 20 was applied to cells for 1 h at room temperature. For Actin: cells were stained for 1 h using SiR-actin (Cytoskeleton, Inc., Denver, CO), or Alexa Fluor™ 555 Phalloidin (Thermo Fisher Scientific), in 1.25 % fish skin gelatin, 2.5 % FBS, 0.5 % triton X-100, 0.25 % tween 20. For all preparations: cells were washed with PBS, and ProLong™ Diamond Antifade Mountant (Thermo Fisher Scientific) was applied to samples, sealed with a coverslip, and imaged. Mammalian cell nuclei were stained using NucBlue® Live ReadyProbes® Reagent (Thermo Fisher Scientific) with the addition of secondary antibody (microtubules preparation) or SiR-Actin (actin preparation).

Cytoskeleton inhibitors

Nocodazole (Sigma-Aldrich) and latrunculin A (Abcam, Cambridge, UK) were dissolved in DMSO to form stock solutions (10 mg/mL and 240 µM respectively) and stored in aliquots at -20 °C. When needed for experiments, a 10 μg/mL stock solution of nocodazole was made by dilution in 100% ethanol immediately prior to addition to cells, with further dilution in KGM-2 to a final concentration of 100 ng/mL. Latrunculin A was diluted to a final concentration of 0.5 µM in KGM-2 + 1.15 mM calcium. Nocodazole or latrunculin A, or both, were added at 3 h post-inoculation in bacterial internalization, intracellular survival, and exit assays, and for imaging. Both compounds were present throughout the remainder of the assays or imaging.

Electron microscopy

Human corneal epithelial cells were seeded on gridded coverslips (MatTek Corporation, Ashland, MA) and inoculated with *P. aeruginosa* PAO1, *pilA*::Tn, or *pilT*::Tn (containing the T3SS reporter pJNE05 for consistency with other experiments) at an MOI of 10 and incubated for 3 h at 37 ^o^C. After 3 h, extracellular bacteria were killed with amikacin (200 µg/mL) for 3 h at 37 °C. At 6 h, fluorescence images were acquired using a Nikon Ti-E inverted wide-field fluorescence microscope to note localization of cells containing intracellular bacteria. Cells were washed with PBS and fixed for 15 min in 4 % PFA. Samples were further fixed with 2.5 % glutaraldehyde in 0.1 M sodium cacodylate buffer. Samples were post-fixed with 1% osmium tetroxide (Sigma-Aldrich) and 1.6 % potassium ferrocyanide (Sigma-Aldrich), dehydrated in a graded series of ethanol, and embedded in EmBed 812 epon araldite resin (Electron Microscopy Sciences, Hatfield, PA). Samples were trimmed, sectioned, and stained with 2 % uranyl acetate (Electron Microscopy Sciences) and lead citrate (Sigma-Aldrich). Images were acquired on a FEI Tecnai 12 transmission electron microscope (University of California, Berkeley Electron Microscopy Laboratory).

Computational quantification of twitching motility

Time-lapse images were captured on a Nikon Ti-E inverted wide-field fluorescence microscope for a duration of 5 m, with 10 s frame intervals. The moment speed of individual bacterial cells was determined by measuring the peak displacement of individual bacterial cells between pairs of frames in a time-lapse series. Specifically, a region of interest (ROI) around each infected cell were drawn by hand, everything outside the ROI was cleared, and then each frame was binarized to generate a mask of all bacteria with a given frame. A Euclidian distance transform (EDT) was then performed to get the distance from each bacterium. A mask of the bacteria in the next frame was then overlaid onto the current EDT to then measure the peak displacement of each bacterium between frames. Moment speed was then measured for all frame pairs in a given live imaging time-lapse. The 90^th^ percentile of speeds were processed to exclude Brownian motion (movements between 1-2 pixels in length), thresholding noise, and movements of bacteria in the Z-plane. The full analysis code can be found here:

<https://github.com/Llamero/Measure_bacterial_speed-macro>

Statistical analysis

Data were expressed as a mean ± standard deviation except for bacterial velocity which was expressed as a median with upper and lower quartiles. Bacterial internalization and intracellular survival, exit assays, and microtubule comparisons were each analyzed by One-way ANOVA with Dunnett’s multiple comparison test. Electron microscopy image data was analyzed using Fisher’s exact test (one-tailed). GraphPad Prism 6 was used for analysis. Computational quantification of twitching motility was analyzed using a Kruskal-Wallis test and One-sided Wilcoxon test in R (x64 3.4.1). The full R code can be found here:

<https://github.com/Llamero/Measure_bacterial_speed-macro>

*P* values less than, or equal to, 0.05 were considered significant.
